# Zeolite Membranes for Gas and Liquid Separation: Synthesis and Applications

**DOI:** 10.3390/membranes15010024

**Published:** 2025-01-13

**Authors:** Qing Wang, Xiaoyu Yang, Bin Wang

**Affiliations:** 1 School of Energy, Materials and Chemical Engineering, Hefei University, Hefei 230601, China; 2 State Key Laboratory of Materials-Oriented Chemical Engineering, College of Chemical Engineering, Nanjing Tech University, Nanjing 210009, China; binerwong@njtech.edu.cn

The quest for efficient separation technologies is more critical than ever in our rapidly evolving industrial landscape, where the demand for sustainable and cost-effective solutions is paramount [1,2]. Among various separation technologies, zeolitic membranes have emerged as a promising solution for gas and liquid separation applications [3]. Zeolites are microporous materials characterized by unique pore structures and tunable chemical properties, which endow them with molecular sieving and selective adsorption capabilities. The development of zeolite membranes has significantly advanced our understanding of continuous separation processes based on size and affinity, with numerous milestones achieved through research and application. Recent studies have shown substantial progress in synthesizing zeolite membranes using innovative techniques such as hydrothermal synthesis, xerogel formation, interlayer engineering, and post-synthesis modification, all of which enhance their performance.

In gas separation, zeolite membranes have shown great promise in applications such as carbon dioxide capture, hydrogen purification, and the separation of light hydrocarbons. Their ability to selectively allow certain gases to permeate while rejecting others can lead to more efficient separation processes. Furthermore, integrating zeolite membranes into existing systems (i.e., re-engineering conventional processes) can facilitate a smooth transition to greener technologies, in line with global sustainable development goals, and help achieve carbon peak and carbon neutrality [4,5]. Liquid separation applications also benefit from the unique properties of zeolite membranes. Their ability to effectively separate organic–organic solvents and water–organic solvent systems and to recover valuable components from waste streams fully demonstrates the versatility of zeolite membranes.

Therefore, the aim of this Special Issue was to focus on the latest theoretical and research advances related to materials chemistry, membrane synthesis, characterization, simulation, and performance in membrane separation. Topics of interest included, but were not limited to, zeolites, metal–organic frameworks (MOFs), novel membrane materials, innovative membrane construction methods, post-treatment techniques, and membrane processes. This Special Issue features eight compelling papers that advance our understanding and application of membrane technology, focusing on innovative strategies for gas and liquid separation processes. The contributions highlight the synthesis, characterization, and functional performance of various membranes, demonstrating their potential in addressing contemporary separation challenges.

Wang et al. [6] meticulously reconstructed the phase equilibria of the V-Ti-Fe system using the CALculation of PHAse Diagrams method. Their pioneering effort in fabricating a 500 mm long tubular membrane revealed a hydrogen flux of 4.06 mL min^−1^, outperforming traditional plate-like membranes, thus underscoring the importance of structural design in enhancing hydrogen transport. Similarly, Yan et al. [7] contributed significantly by establishing the first phase diagram for the V-Ti-Co system, addressing a crucial gap in the literature. Their findings revealed a peak permeability of 4.05 × 10^−8^ mol H_2_ m^−1^ s^−1^ Pa^−0.5^ for specific V-Ti-Co alloys, showcasing the importance of composition on hydrogen transport behavior. The authors attributed this high permeability to the synergistic effects of increased hydrogen solubility and diffusivity, providing valuable insights for future alloy development. Ivanov et al. [8] utilized dielectric spectroscopy to explore dielectric relaxations in erythrocyte membranes, revealing critical insights into the mechanical properties and inter-membrane interactions under varying ionic conditions. This research lays the groundwork for understanding how membrane flexibility affects transport phenomena in biological systems.

Ding et al. [9] presented a novel approach to enhance the desalination stability of GO-EDA/Al_2_O_3_ tubular nanofiltration membranes by optimizing the pH of the GO-EDA suspension. Their GE-11 membrane achieved a rejection rate of 91.5% for 1 mM Na_2_SO_4_ at 5 bar after soaking in water for 680 h, increasing to 96.3% at 20 bar. This work highlights the potential of charge manipulation in developing durable GO-derived nanofiltration membranes for water treatment. Herzog et al. [10] investigated diffusion barriers to minimize strength degradation in reactive air-brazed BSCF membranes during aging. They found that applying diffusion layers to AISI 314 steel significantly improved the bending strength of BSCF joints from 17 MPa to 35 MPa after aging at 850 °C for 1000 h. These results offer insights that could benefit other joining systems in high-temperature applications. Zhao et al. [11] introduced a highly flexible ZIF-8 membrane with exceptional lithium-ion separation capabilities. They used a dopamine-assisted co-deposition technique to introduce numerous hydroxyl and amine groups on the MPPM surface, providing heterogeneous nucleation sites for ZIF-8 growth. Their membrane achieved a lithium-ion permeation flux of 0.151 mol m^−2^ h^−1^ and exhibited impressive selectivity, crucial for energy storage applications. Notably, the membrane retained its performance under mechanical stress, highlighting the importance of flexibility in membrane applications.

Tong et al. [12] reported a novel approach to enhance ethanol dehydration via efficient pervaporation using polyvinyl alcohol (PVA)/Ti_3_C_2_T_x_ nanosheet mixed matrix membranes. Their membranes exhibited remarkable performance, achieving a water flux of 1.21 kg·m^−2^·h^−1^ and a separation factor of 1126.8, attributed to enhanced hydrophilicity and diffusion channels provided by the MXene (Ti_3_C_2_T_x_). With excellent mechanical strength and stability, these membranes maintained performance over extended testing, promising significant energy savings and enhanced efficiency in ethanol dehydration applications. Lastly, Zhou et al. [13] reported on the in situ incorporation of TiO_2_@GO nanosheets within polyacrylonitrile (PAN) membranes for ultrafast protein separation. The hybrid membrane exhibited a remarkable water flux of 1487.6 L·m^−2^·h^−1^, paired with a rejection rate of 99.5% for bovine serum albumin. Their work showcased the synergy between hydrophilic TiO_2_ nanoparticles and graphene oxide, which enhanced both flux and anti-fouling properties, thereby expanding the potential applications of ultrafiltration membranes in bioprocessing.

This Special Issue aims to illuminate the latest advancements in membrane technology, showcasing innovative approaches and significant contributions in the field. The findings presented herein not only deepen our understanding of membrane materials and their applications but also provide valuable insights into the synthesis and optimization of these materials.
